# Potential Misinterpretation of Treatment Effects Due to Use of Odds Ratios and Logistic Regression in Randomized Controlled Trials

**DOI:** 10.1371/journal.pone.0021248

**Published:** 2011-06-16

**Authors:** Mirjam J. Knol, Ruben G. Duijnhoven, Diederick E. Grobbee, Karel G. M. Moons, Rolf H. H. Groenwold

**Affiliations:** Julius Center for Health Sciences and Primary Care, University Medical Center Utrecht, Utrecht, the Netherlands; University College London - Institute of Child Health, United Kingdom

## Abstract

**Background:**

In randomized controlled trials (RCTs), the odds ratio (OR) can substantially overestimate the risk ratio (RR) if the incidence of the outcome is over 10%. This study determined the frequency of use of ORs, the frequency of overestimation of the OR as compared with its accompanying RR in published RCTs, and we assessed how often regression models that calculate RRs were used.

**Methods:**

We included 288 RCTs published in 2008 in five major general medical journals (Annals of Internal Medicine, British Medical Journal, Journal of the American Medical Association, Lancet, New England Journal of Medicine). If an OR was reported, we calculated the corresponding RR, and we calculated the percentage of overestimation by using the formula 
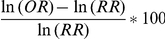
.

**Results:**

Of 193 RCTs with a dichotomous primary outcome, 24 (12.4%) presented a crude and/or adjusted OR for the primary outcome. In five RCTs (2.6%), the OR differed more than 100% from its accompanying RR on the log scale. Forty-one of all included RCTs (n = 288; 14.2%) presented ORs for other outcomes, or for subgroup analyses. Nineteen of these RCTs (6.6%) had at least one OR that deviated more than 100% from its accompanying RR on the log scale. Of 53 RCTs that adjusted for baseline variables, 15 used logistic regression. Alternative methods to estimate RRs were only used in four RCTs.

**Conclusion:**

ORs and logistic regression are often used in RCTs and in many articles the OR did not approximate the RR. Although the authors did not explicitly misinterpret these ORs as RRs, misinterpretation by readers can seriously affect treatment decisions and policy making.

## Introduction

A randomized controlled trial (RCT) is generally considered the best approach to estimate treatment benefits. Results of RCTs are used to make treatment decisions for clinical practice and policy making. If the outcome of a RCT is dichotomous, the treatment effect can be expressed by several effect estimates, e.g. the risk difference, the risk ratio (RR), the odds ratio (OR), and the hazard ratio which is used for time-to-event data. The OR is commonly interpreted as a RR. As long as the incidence of the outcome is not too high, e.g. below 10%, the OR is a good approximation of the RR. However, if the incidence is high, the OR can substantially overestimate the RR [1], [2]. In addition, the further away the OR is from 1, the larger the overestimation. We note that in case-control studies, in contrast to RCTs and cohort studies, the outcome does not need to be rare for the OR to approximate the RR in certain situations [3].

As an example of overestimation of the RR by the OR in a RCT, suppose that 500 patients are treated with drug A and 500 with placebo. The outcome is survival for 30 days. In the treatment arm 85% of the patients survive 30 days, and in the placebo arm 70% of the patients survive 30 days. The OR for survival would be 2.4, and the RR 1.2 (see Table 1). If the OR is interpreted as a RR, the conclusion would be that patients using drug A have a 2.4 times higher chance to survive than patients using placebo, while in reality patients using drug A have only 1.2 times higher chance of survival than placebo users. Another well known example is by Schulman *et al.* who presented an OR of 0.6 of referral for cardiac catheterization in blacks as compared with whites, and for women as compared with men, while the RR was only 0.93 [4], [5]. A newspaper report stated about this research: “Blacks and women with chest pain are 40% less likely than whites or men to be referred by physicians for cardiac catheterization”, while this was only 7% [5]. Interpreting ORs as RRs can thus lead to erroneous conclusions, which could seriously affect treatment decision making.

**Table 1:** Suppose that 500 patients are treated with drug A and 500 with placebo.

	Survival after 30 days		
	Yes (N, %)	No (N, %)	Total
**Drug A**	425 (85%)	75 (15%)	500
**Placebo**	350 (70%)	150 (30%)	500
**Total**	775	225	1000

The outcome is survival for 30 days. In the treatment arm 85% of the patients survive 30 days, and in the placebo arm 70% of the patients survive 30 days. The two-by-two table looks as above.

The odds ratio is calculated as the ratio of the odds of treatment in the patients who survived (425/350 = 1.21) and the odds of treatment in the patient who did not survive (75/150 = 0.50), resulting in an odds ratio of 1.21/0.50 = 2.43. One could also calculate the cross-product of the table: (425*150)/(350*75) = 2.43.

The risk ratio is calculated as the ratio of the risk of survival in the treatment group (425/500 = 0.85) and the risk of survival in the placebo group (350/500 = 0.70), resulting in a risk ratio of 0.85/0.70 = 1.21.

Researchers often present ORs to quantify the treatment effect in a RCT, because they have applied logistic regression to adjust for baseline covariables. Logistic regression models yield odds ratios. However, other multivariable regression models that directly estimate RRs are available, such as log-binomial regression [6] and Poisson regression with robust standard errors [7].

The aim of this study was to investigate the potential for misinterpretation of treatment effects in a sample of published RCTs by determining the frequency of use of ORs, and the frequency of overestimation of the OR as compared with its accompanying RR. In addition, we assessed how often regression models that directly calculate RRs were used. In the end, we give three illustrative examples from the included RCTs to highlight that misinterpretation of odds ratios can occur and that we can do better in analyzing trials.

## Methods

### Selection of articles

We included all RCTs published in 2008 in five major general medical journals (Annals of Internal Medicine, British Medical Journal, Journal of the American Medical Association, Lancet, and New England Journal of Medicine). We identified these RCTs in a search on Pubmed combining the journal names with the publication type ‘randomized controlled trial’ and publication date ‘2008’. RCTs that were published online in 2008 but on paper in 2009 were excluded. Furthermore, letters and studies that were not RCTs were excluded.

### Data extraction

A standardized data extraction form was used to assess the articles. Two reviewers (MK and RG) independently extracted the data, each of them assessing half of the articles. We extracted general items including journal name, number of subjects, type of treatment, and the scale of the primary outcome (continuous, dichotomous, ordinal, count). For articles with a dichotomous primary outcome we extracted the crude measure of effect of the primary outcome (e.g. OR, RR), the analysis that was used to adjust for baseline variables if applicable (e.g. logistic regression, Cox regression), and the adjusted measure of effect of the primary outcome if applicable (e.g. OR, hazard ratio). In addition, we extracted whether any other OR was mentioned in the articles, for example for secondary outcomes or subgroup analyses. If ORs were presented, we also extracted the incidence of the outcome in the non-exposed group to calculate the amount of overestimation of the OR as compared with the RR (see below). If RCTs performed stratified randomization and adjusted for these stratification factors in the analysis, we did not score this as adjustment for baseline variables. We did not extract any ORs that were part of a prognostic prediction model made with the RCT data, because in this article we were interested in estimation of treatment effects.

### Data analysis

Frequencies and summary statistics of the extracted items were presented. If an OR was reported and the incidence in the non-exposed group was known, we calculated the corresponding RR to estimate the extent of overestimation of the OR, using the formula: 
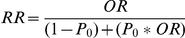
, where P_0_ is the incidence of the outcome in the non-exposed group [2]. Subsequently, we calculated the percentage difference between the presented OR and calculated RR by using the formula: 
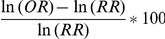
. The formula to calculate RRs based on the OR and the incidence of the outcome in the non-exposed has been criticized, especially for adjusted ORs [8], [9]. The RR calculated using this formula gives some bias away from the null, and this increases with increasing outcome incidence and effect estimate. This means that we slightly underestimate the percentage difference between the presented OR and calculated RR.

## Results

### Characteristics of the included RCTs

Our primary search yielded 349 articles. We excluded 8 articles that were published online in 2008 but on paper in 2009, 31 letters, and 22 articles that were not RCTs. This resulted in 288 RCTs that were published in Annals of Internal Medicine (n = 16, 5.6%), BMJ (n = 45, 15.6%), JAMA (n = 51, 17.7%), Lancet (n = 80, 27.8%), and New England Journal of Medicine (n = 96, 33.3%). Most RCTs investigated the effect of drugs (64.2%). The median number of patients included in the RCTs was 663 with a range of 13 to 31,290. The majority of the RCTs had a dichotomous primary outcome (n = 193, 67.0%), while 30.6% and 2.4% had a continuous and other outcome (e.g., count, ordinal), respectively.

### Use of ORs for primary outcome

**Figure 1:**
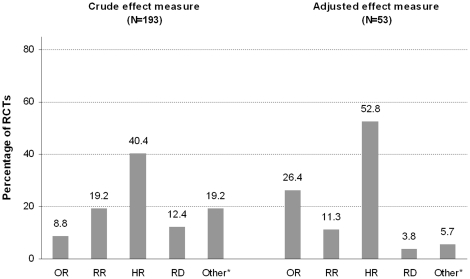
Crude effect measures presented in 193 RCTs that had a dichotomous primary outcome, and adjusted effect measures presented in 53 RCTs that had a dichotomous primary outcome and adjusted for baseline variables (OR = odds ratio, RR = risk ratio, HR = hazard ratio, RD = risk difference). * Other includes presentation of only p-values or only percentages, or no crude measure presented.

**Figure 2:**
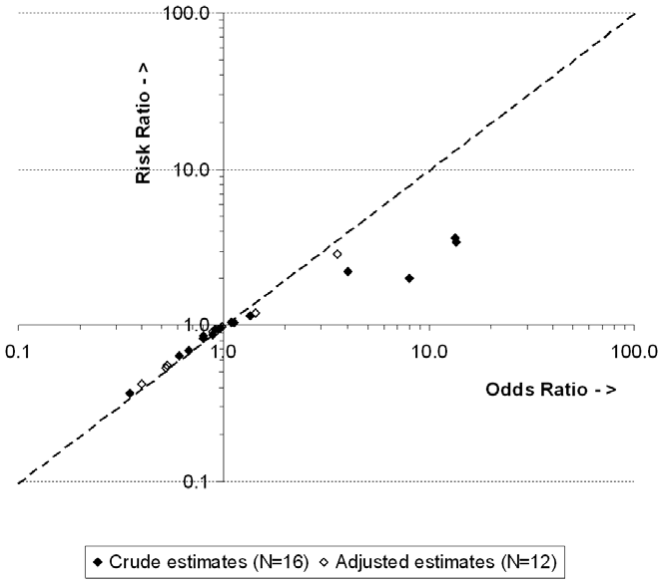
ORs and their accompanying RRs for the primary outcome in 16 RCTs (crude estimate) and 12 RCTs (adjusted estimate). Dotted line represents the points where the OR and RR are the same.

Of the 193 studies with a dichotomous primary outcome, the majority presented hazard ratios because the outcome was time-to-event (Figure 1). RRs were also often presented as crude effect estimates (19.2%) but less as adjusted effect estimates (11.3%). Ten RCTs only presented a crude OR, 7 only presented an adjusted OR, and 7 presented both a crude and adjusted OR, so a total of 24 RCTs (12.4%) presented a crude and/or adjusted OR for the primary outcome. Figure 2 presents the crude and adjusted ORs and their accompanying crude and adjusted RRs calculated using the formula above (for three ORs the accompanying RR could not be calculated because the incidence of the outcome was not given (1×) and because the value of the adjusted OR was not given (2×)). In six RCTs (3.1%) the OR differed between 50% and 100% from the RR on the log scale, and in five RCTs (2.6%) the OR differed more than 100% from the RR on the log scale (e.g. OR = 8.0, RR = 2.0). In the latter five RCTs, the overall incidence of the outcome ranged from 53–64%.

None of the 24 RCTs that presented ORs explicitly interpreted the OR as a RR, for example stating that the risk of the outcome was doubled in the treatment group as compared to the control group when the OR is 2.0. However, three RCTs stated that the risk of the outcome was lower/greater while reporting an OR between brackets after this statement, suggesting that the OR could be interpreted as a RR, which in one RCT was obviously not the case (OR = 13.40, RR = 3.66). Furthermore, none of the RCTs that presented ORs warned the reader that the OR could not be interpreted as a RR.

### Use of other ORs than for primary outcome

**Figure 3:**
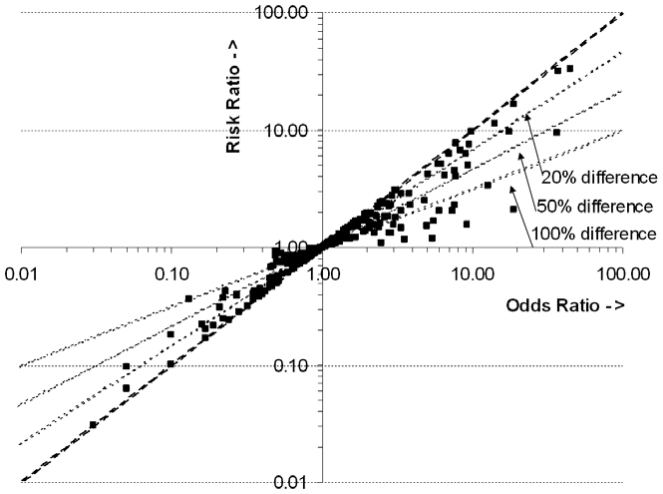
ORs and their accompanying RRs for other outcomes or subgroup analyses in 41 RCTs. Dotted line represents the points where the OR and RR are the same; other lines represent 20%, 50% and 100% difference between OR and RR.

Forty-one of all included RCTs (14.2%) presented ORs for other outcomes than the primary outcome, or for subgroup analyses. Nineteen RCTs (6.6%) had at least one OR that deviated more than 100% from its accompanying RR on the log scale (e.g. OR = 5.4, RR = 1.2; OR = 0.49, RR = 0.94) (Figure 3). The ORs that showed more than 100% deviation had accompanying overall incidences of the outcome from 50 to 92%.

### Use of alternative methods

Among the 193 RCTs with a dichotomous primary outcome, 53 (27.5%) adjusted for baseline variables. Twenty-six RCTs (13.5%) used Cox regression because the outcome was time-to-event. Fifteen RCTs (7.8%) used logistic regression. Alternative methods for logistic regression to directly estimate RRs were only used in four RCTs (2.1%): two used Poisson regression with a robust standard error, one used log-binomial regression and one used the Mantel-Haenszel procedure. Three RCTs used other methods including negative binomial regression, generalized linear mixed model with a log link, and generalized estimating equations, and in five RCTs the method for adjustment was unclear.

### Illustrative examples

From the included RCTs we took three illustrative examples to highlight that misinterpretation of ORs can occur and that we can do better in analyzing trials.

#### Example 1

Morley *et al.* [10] studied whether nasal continuous positive airway pressure (CPAP), rather than intubation and ventilation, shortly after birth reduces the rate of death or bronchopulmonary dysplasia in very preterm infants. In Table 2 and Table 3 of the original article, the authors present many ORs for different outcomes and subgroups. Most outcomes that were studied were common, e.g. the primary outcome death or oxygen treatment at 36 weeks gestational age occurred in 36.4% of the infants. One of the most striking examples of large deviation between OR and RR in this study was for the outcome death, oxygen treatment, or respiratory support at 28 days of age in the subgroup with infants of 25 or 26 weeks gestation (see Table 3 in original article). In infants receiving CPAP the outcome occurred in 89.0% and in infants receiving intubation and ventilation the outcome occurred in 94.3%, resulting in an OR of 0.49 (95% CI: 0.17–1.39). However, the corresponding RR is only 0.94 (95% CI: 0.87–1.03). Readers misinterpreting the OR as a RR could conclude that infants on CPAP have a 51% reduced risk of the outcome, while in reality this is only 6%.

#### Example 2

Wheeler *et al.* [11] studied whether expression of drug concentration as a ratio (1 ml of a 1∶1000 solution) instead of mass concentration (1 mg in 1 ml) on epinephrine ampules caused dosing errors. In the ratio group 11 of the 14 doctors made a dosing error and in the mass concentration group 3 of the 14 doctors made a dosing error. The authors presented an OR: 13.4 (95% CI: 2.2–81.7). However, the corresponding RR is only 3.7 (95% CI: 1.3–10.4).

#### Example 3

Smolen *et al.* [12] studied the effect of tocilizumab on 20% improvement of signs and symptoms of rheumatoid arthritis. At 24 weeks, response was higher in patients receiving tocilizumab 8 mg/kg (120/205 = 59%) than in patients receiving placebo (54/204 = 26%). The authors presented an OR adjusted for region of 4.0 (95% CI: 2.6–6.1). However, using the formula 
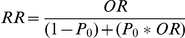
, the corresponding RR is only 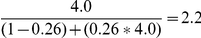. Because the outcome in this study is common, the OR and RR deviate substantially. If a reader interprets this OR as a RR, he would conclude that patients taking tocilizumab 8 mg/kg have a four times higher probability of having a good response than patients taking placebo, while in reality this probability is only 2.2 times higher.

## Discussion

This review showed that, despite its difficult interpretation, the OR is a popular effect measure in RCTs as one-in-eight RCTs with a dichotomous outcome presented an OR for the primary outcome, and one-in-seven of all RCTs presented any OR. Moreover, the OR often overestimated the RR by more than 20% on the log scale. Furthermore, after Cox regression, logistic regression was the most often used method to perform baseline adjustment in RCTs with dichotomous outcomes. Alternative models to calculate RRs were almost never used.

Although the OR can seriously overestimate the RR, we found that it is still often estimated in RCTs, even for crude analyses. A reason may be that an OR has some mathematical advantages over a RR, including its symmetry with respect to ‘successes’ and ‘failures’ and the fact that the OR may assume values unrestricted between zero and infinity [13]. In addition, the use of ORs itself is not wrong as long as the interpretation is correct, i.e. in the case of a RCT, the odds for the outcome in the treated group (over a certain time period) as compared with the odds for the outcome in the control group. We found that none of the authors explicitly interpreted the OR as RR. However, policy makers or the media can easily misinterpret ORs as RRs, the latter being nicely illustrated by Schwartz *et al* [5]. Our survey showed that substantial overestimation was present in one third (for primary outcomes) to two thirds (for secondary outcomes or subgroup analyses) of RCTs that present ORs, potentially leading to misinterpretation of results.

Although various alternatives for logistic regression are available, apparently they are not used for adjustment for covariables in RCTs. There are several possible reasons for this. First, it may not be known to authors of clinical papers that the OR can overestimate the RR. This knowledge could be enhanced by education and publishing articles on this issue in clinical papers. Second, authors are probably more familiar with logistic regression and do not know that there are alternative methods to estimate adjusted RRs. This could be partly due to the fact that most literature on the methods and use of these alternative methods is published in epidemiology journals rather than clinical journals. Third, from a statistical point of view logistic regression is convenient for dichotomous outcomes as the predicted probabilities are restricted between 0 and 1. However, simulation studies have shown that alternative models behave well with regard to bias and coverage of the 95% confidence interval [9], [14] and seem not to be influenced by possible misspecification of the model. Further research should be done to assess whether these positive results apply to all situations and datasets. All widely used statistical software packages have the possibility to fit models such as Poisson regression with robust standard errors and log-binomial regression [15], [16], which should therefore not be a barrier to use these models. The use of alternative models could be increased by publication of papers on methods and applications of these models in clinical journals. Also reviewers and editors of clinical journals should be aware of the possibilities to use alternative models. Lumley et al. and McNutt et al. nicely summarized several methods and showed how to apply the methods [9], [15].

Our study was based on articles published in leading medical journals and our results may therefore not be applicable to RCTs published in less prominent journals. Other studies are needed to investigate if there are certain medical specialties that are at a higher risk for misinterpretation of results due to the use of ORs. Despite the fact that we included a large number of RCTs in our study, we ended up with a relatively low number of RCTs that had a dichotomous outcome and did not analyze their data as time-to-event. Our study should be repeated in a larger sample to get more robust results. We may have missed some RCTs in our Pubmed search. However, there is no reason to assume that we selectively missed particular RCTs so this will most likely not have influenced our results. We estimated the extent of overestimation of the OR using the formula of Zhang and Yu [2]. The RR calculated using this formula gives some bias away from the null [8], [9], and this increases with increasing outcome incidence and effect estimate. This means that we slightly underestimated the percentage difference between the presented OR and calculated RR. So the extent of overestimation of the RR by the OR may even be greater than we presented. In our study we focused on the misinterpretation of the OR. However, also hazard ratios, which we found are commonly used in RCTs, are difficult to interpret and alternatives to hazard ratios have been proposed [17]–[20]. Moreover, there is elaborate discussion on whether relative or absolute measures of effect are most informative, but this discussion is out of the scope of this paper.

In conclusion, ORs and logistic regression are often used in RCTs currently published in high impact journals. In a substantial number of articles the OR did not adequately approximate the RR. Misinterpretation of ORs by authors, readers or media can seriously affect treatment decisions and policy making. Further research is needed to study the real impact of using ORs on reader's interpretation. We recommend presenting RRs rather than ORs in RCTs, and using alternative models to calculate RRs directly if adjustment for baseline variables is considered necessary to avoid misinterpretation by readers, media or policy makers.
